# Data-driven theoretical characterization of β-decay spectra in radioisotope energy materials via artificial fish swarm optimized adaptive kernel density estimation

**DOI:** 10.3389/fchem.2026.1834067

**Published:** 2026-05-08

**Authors:** Yichen Ma, Changlong Cai, Huachen Liu, Hongmei Zong, Xuerui Wang, Chenxing Wu

**Affiliations:** 1 School of Optoelectronic Engineering, Xi’an Technological University, Xi’an, China; 2 School of Armament Science and Technology, Xi’an Technological University, Xi’an, China

**Keywords:** adaptive kernel density estimation, artificial fish swarm optimization, beta decay energy spectrum, probability density function estimation, root mean square error

## Abstract

**Introduction:**

Accurate modeling of beta-decay energy spectra is essential for theoretical analysis and performance optimization in radioisotope energy conversion. Conventional parameterization methods may introduce fitting bias, while fixed-bandwidth KDE lacks local adaptability for asymmetric, multi-peak and long-tailed spectral distributions.

**Methods:**

This study developed an Artificial Fish Swarm Algorithm-Adaptive Kernel Density Estimation (AFSA-AKDE) framework. After spectral data preprocessing, RMSE was used as the fitness function, the bandwidth was reformulated as an adaptive coefficient-controlled structure, and AFSA was used to optimize key parameters through foraging, swarming and following behaviors.

**Results:**

The method was validated using Ni-63, S-35 and C-14 samples with different thicknesses. For a 2 um Ni-63 sample, AFSA-AKDE achieved MAE = 0.0116%, RMSE = 0.0156% and R2 = 0.9998792. For a 0.2 um C-14 sample, it achieved MAE = 0.0158%, RMSE = 0.0243% and R2 = 0.996687.

**Discussion:**

Compared with fixed-bandwidth KDE, normal distribution models and recent adaptive KDE variants, AFSA-AKDE provides improved accuracy, stability and local adaptability for reconstructing complex beta-decay energy distributions.

## Introduction

The β-decay energy spectrum characterizes the energy distribution characteristics of radioactive particles and is a key foundation for nuclear battery modeling and radiation detection analysis. The accuracy of energy spectrum modeling directly affects the results of particle transport simulation and energy deposition calculation, thus affecting the reliability of related system design (Canning et al., 2023). Existing modeling processes suffer from accumulated fitting errors and distorted distribution representations, limiting the accurate characterization of complex energy spectrum structures (Acero et al., 2024). Research on the high-precision reconstruction of the energy spectrum probability density function has clear theoretical value and engineering significance (Kijim et al., 2024).

The β-decay energy spectrum has asymmetric, multi-peak, and long-tailed distribution characteristics, with a complex statistical structure, making it difficult for traditional parametric models to fit stably. Although nuclear density estimation escapes the limitations of distribution assumptions, bandwidth selection is sensitive to local structures, easily leading to oversmoothing or overfitting (Bagkavos et al., 2026; Yu et al., 2022). Energy spectrum data is affected by material thickness and particle transport processes, exhibiting significant non-stationarity, making it difficult for unified modeling strategies to adapt to the needs of multiple scenarios (Hışıroğlu et al., 2025; Kavun et al., 2025). The lack of an effective coordination mechanism between the global and local bandwidth parameters has become a key factor restricting model accuracy (Ameijeiras-Alonso, 2024; Tenreiro, 2022).

To address the above problems, related research has explored nonparametric estimation and parameter optimization. Adaptive bandwidth-based kernel density estimation methods introduce density-related weights to adjust the degree of local smoothness, demonstrating high fitting ability in complex distribution modeling (Reyes et al., 2017; Tenreiro, 2020). For example, Zhao W and Tabak (Zhao and Tabak, 2025) proposed a factor-dependent bandwidth construction method, which drives the adaptive adjustment of bandwidth through the effective number of samples and variance, and extends it to the case of categorical variables to integrate cross-class information and intra-class features, thereby improving the accuracy of conditional density estimation; optimization methods for bandwidth selection problems gradually introduce swarm intelligence algorithms, improve the stability of parameter optimization through global search mechanisms, and avoid getting trapped in local optima (Jin et al., 2021; Pelz et al., 2023). For example, Zámečník et al. (Zámečník et al., 2024) proposed an adaptive bandwidth selection strategy for circular kernel density estimation, construct a variable bandwidth mechanism in multimodal and skewed distribution scenarios, and verify the stable performance of the bandwidth adjustment strategy under complex distributions through simulation and real data; some studies combine kernel density estimation with filtering or regression frameworks to enhance the robustness of the model to abnormal data and noise interference (Grooms and Riedel, 2024; Riedel et al., 2025). For example, Wang et al. (Wang et al., 2025) constructed a nonparametric adaptive kernel density estimation denoising model, determined the local sample distribution through the K-nearest neighbor structure, determined the search ellipse direction by combining principal component analysis and established a bandwidth estimation mechanism, and introduced a local bias factor and adaptive threshold to achieve noise suppression and signal extraction. Existing methods still have shortcomings in the coordination of bandwidth dynamic adjustment and global optimization, and a unified mechanism has not yet been formed between local fitting accuracy and global consistency.

To address the problem of insufficient coordination between kernel density estimation bandwidth adjustment and parameter optimization, this paper constructs an adaptive kernel density estimation model driven by artificial fish swarm optimization. Initialization is completed by reading β-decay energy spectrum sampling data, Gaussian kernel function is embedded into the density estimation framework and bandwidth parameterization is established, and the bandwidth is reconstructed into a function form with adjustment coefficients. The objective function is constructed based on the error between the kernel density estimation result and the histogram density, and the artificial fish swarm algorithm is introduced to perform parameter optimization. Individual positions are updated and fitness is adjusted synchronously through foraging, swarming and tail chasing behaviors, driving the parameters to converge iteratively in the solution space. The adaptive bandwidth is calculated based on the optimization results, a differentiated smoothing scale is assigned to each sample point and the kernel function is weighted and superimposed to achieve the reconstruction of the probability density function. The method couples a bandwidth adaptive mechanism and a swarm intelligence optimization strategy within a unified framework, forming a highly consistent modeling path for complex energy spectrum distributions. Based on the above modeling process, this paper further systematically designs related issues at the method structure and parameter mechanism levels, resulting in the following research contributions:

1. Constructing a unified modeling framework for artificial fish swarm optimization and adaptive kernel density estimation, coupling the functional expression of bandwidth parameters with the swarm intelligence global search process, forming a co-evolutionary mechanism for bandwidth adjustment and parameter optimization.
2. Introducing a dynamic bandwidth allocation strategy based on local sample density, embedding density-related information into the kernel function smoothing scale adjustment process, enhancing the fine-grained characterization capability for asymmetric, multi-peak, and long-tailed energy spectrum structures.
3. Establishing a probability density modeling process for β-decay energy spectra, systematically integrating data preprocessing, parameter optimization, and density reconstruction, forming a unified nonparametric modeling path applicable to multiple materials and thickness conditions.

### Adaptive kernel density estimation based on artificial fish swarm optimization

#### Adaptive kernel density estimation

The non-parametric KDE method has the advantage of being unaffected by the choice of prior models, thus emerging as a promising research direction in modern statistics and providing theoretical support for data modeling of unknown distribution characteristics and missing data processing in multiple fields (JONES, 1995; Yandell, 1997). Its core idea is to realize the predictive estimation of probability density using the samples themselves, without imposing any assumptions on the distribution. If the density function of the random variable X is $f\left(x\right)$ , and since $f\left(x\right)=F^{\prime }\left(x\right)$ , according to the basic idea of kernel density estimation, a simple estimator of f can be obtained, namely,:

$$f_{n}\left(x\right)=\frac{F\left(x+h\right)+F\left(x‐h\right)}{2h}$$ (1)

In the formula, $h=h\left(n\right)$ is a strictly positive constant; n is the total number of samples to be estimated; $F\left(x\right)$ is the function of the empirical distribution of the random variable X .

When selecting h such that n→+∞ , h→0 and the product nh is maintained at a constant positive value *C*, then nh→+∞ as *n* increases, which ensures the estimator consistency, the estimated density function $f\left(x\right)$ can have good properties. The estimator of the kernel density function is as follows:

$$\hat{f}\left(x\right)=\frac{1}{nh}\sum_{i=1}^{n}K\left(\frac{x‐x_{i}}{h}\right)$$ (2)

Where: $\hat{f}\left(x\right)$ is the energy kernel density estimation function based on non-parametric KDE; $K\left(\cdot \right)$ is the kernel function; h is the bandwidth; $x_{i}$ is the i-th energy sample value; n is the total number of samples.

Under a determined bandwidth h , to avoid the kernel function $K\left(\cdot \right)$ from causing discontinuous effects on the probability density function, the following constraint needs to be set (Yang et al., 2019), where c is a constant:

$$\left\{\begin{array}{c} \&\int K\left(\cdot \right)dx=1\\ \&\int xK\left(\cdot \right)dx=0\\ \&\int x^{2}K\left(\cdot \right)dx=c \end{array}\right.$$ (3)

There are many forms of the kernel function $K\left(\cdot \right)$ , among which the Gaussian kernel function, which has good mathematical properties, is the most cited. When $K\left(\cdot \right)$ is in the form of Equation 3, under the selection of different kernel functions, the model fitting error of the non-parametric KDE is in a small fluctuation range. It can be considered that, ignoring such errors, using the Gaussian function as the kernel function for probability density estimation has a good fitting effect (EPANECHNIKOV, 1969), that is:

$$K\left(x\right)=\frac{1}{\sqrt{2\pi }}e^{\left(‐\frac{x^{2}}{2}\right)}$$ (4)

Combining Formula 4 and Formula 1, the non-parametric KDE of the energy probability density function can be rewritten as:

$$\hat{f}\left(x\right)=\frac{1}{\sqrt{2\pi }nh}\sum_{i=1}^{n}e^{{\left[‐\frac{1}{2}\left(\frac{x‐x_{i}}{h}\right)\right]}^{2}}$$ (5)

Where: $\hat{f}\left(x\right)$ is the energy kernel density estimation function based on non-parametric KDE; $K\left(\cdot \right)$ is the kernel function; h is the bandwidth; $x_{i}$ is the i-th energy sample value; n is the total number of samples.

In KDE, the selection of bandwidth determines the smoothness of the fitted curve: the larger the bandwidth, the smoother the curve, but the poorer the fitting effect. Currently, there are numerous methods for determining fixed bandwidth, such as the empirical rule, cross-validation, penalty function method, and comparison methods. Among these, the empirical rule is based on the idea of minimum squared error and solves for the optimal bandwidth by minimizing the mean integrated square error (MISE) (Se et al., 2014); this method is the most commonly used. When its kernel function is specified as the Gaussian kernel, the formula is:

$$h\approx 1.06\hat{\sigma }n^{‐\frac{1}{5}}$$ (6)

In Formula 6, h is the fixed bandwidth determined using the empirical rule, and $\hat{\sigma }$ is the standard deviation of the sample. The KDE models obtained through fixed bandwidth all have the issue of low local goodness of fit. Therefore, after calculating the fixed bandwidth, the bandwidth h should be corrected in a targeted manner. The specific improvement strategies are as follows. Based on the kernel density estimation value $\hat{f}\left(x_{i}\right)$ obtained from formula 2, the bandwidth coefficient is designed on the basis that $h_{i}$ is proportional to $\hat{f}{\left(x_{i}\right)}^{‐\frac{1}{2}}$ as shown in Equation 7 (Wenbin et al., 2022):

$$\lambda_{i}={\left[\frac{\hat{f}\left(x_{i}\right)}{g}\right]}^{‐\alpha }$$ (7)

where $g=\frac{1}{n}\sum_{i=1}^{n}\hat{f}\left(x_{i}\right)$ is the arithmetic mean of $\hat{f}\left(x_{i}\right)$ , which has a better effect than the geometric mean (Yin, 2018), α is a sensitivity parameter that satisfies 0≤α<1 . Studies have shown that in practical applications, the effect is the best when α=0.5 . The fixed bandwidth is selected as the initial bandwidth $h_{i}$ through the empirical rule of Formula 6. Therefore, the adaptive bandwidth is ${h_{i}}^{*}=\lambda_{i}h_{i}$ . By replacing $h_{i}$ in Formula 5, the formula of the adaptive kernel density estimation (A-KDE) algorithm can be obtained as:

$${\hat{f}}^{*}\left(x\right)=\frac{1}{\sqrt{2\pi }nh^{*}}\sum_{i=1}^{n}e^{{\left[‐\frac{1}{2}\left(\frac{x‐x_{i}}{h^{*}}\right)\right]}^{2}}=\frac{1}{\sqrt{2\pi }n\lambda_{i}h_{i}}\sum_{i=1}^{n}e^{{\left[‐\frac{1}{2}\left(\frac{x‐x_{i}}{\lambda_{i}h_{i}}\right)\right]}^{2}}$$ (8)

#### Artificial fish swarm optimization

Based on Formula 8 and according to Formula 6, a new bandwidth formula is given: $\hat{h}=c\hat{\sigma }n^{‐\alpha }$ , where c and α are to be determined. The artificial fish swarm algorithm is used to optimize c and α. Taking the kernel density estimation function as the objective function, assuming an N-dimensional space, the artificial fish swarm $X=\left(X_{1},X_{2},\ldots ,X_{m}\right)$ is composed of multiple energy data, where the i-th fish data $X_{i}={\left(X_{i1},X_{i2},\ldots ,X_{iN}\right)}^{T}$ is calculated by the objective function ${\hat{f}}^{*}\left(x\right)$ , resulting in $F\left(X_{i}\right)=\left[{\hat{f}}^{*}\left(x_{i1}\right),{\hat{f}}^{*}\left(x_{i2}\right),\ldots ,{\hat{f}}^{*}\left(x_{iN}\right)\right]$ as a set of potential solutions for the kernel density estimation function. The RMSE is used as the fitness function. In the initial solution, the parameters c and α are set to 1.06 and 0.2. According to the optimization algorithm, the new window width formula is: ${\hat{h}}_{opt}=c_{opt}\hat{\sigma }n^{‐\alpha_{opt}}$ .

The Artificial Fish Swarm Optimization Algorithm (AFSO) is a swarm intelligence optimization algorithm that simulates the foraging, swarming, and following behaviors of fish swarm in nature. The computational cost of the AFSO optimization process is determined by the swarm size, the number of iterations, and the sample size involved in kernel density estimation. In each iteration, the fitness evaluation requires computing the kernel density estimation over all samples, resulting in a computational cost that increases with the square of the sample size. The overall computational cost is therefore determined by the combined effect of swarm size, iteration number, and sample size. To evaluate computational efficiency, the execution time per iteration was measured under different sample sizes, providing a quantitative assessment of the algorithm scalability. To ensure the convergence behavior of the AFSO optimization process, the evolution of the fitness value across iterations was monitored under fixed experimental conditions. The results show that the RMSE value decreases monotonically during early iterations and gradually stabilizes after a finite number of iterations, indicating that the algorithm reaches a steady optimization state without oscillatory divergence. By monitoring the optimization process under 20 different random initial parameters, the difference in the final convergence value of RMSE for all groups was less than 1e-5, and all converged to the same minimum region, which verified that the algorithm achieved stable empirical convergence rather than local optima. In addition, parameter sensitivity analysis was conducted with respect to the fish swarm size and the maximum number of iterations. The swarm size was varied from 10 to 100 with a step of 10, while the iteration number ranged from 50 to 500. The experimental results demonstrate that when the swarm size is below 30, the optimization process exhibits noticeable fluctuations in RMSE, while increasing the swarm size beyond 60 leads to stable convergence with marginal improvement. Similarly, when the number of iterations is less than 100, the optimization results remain unstable, whereas convergence is consistently achieved when the iteration number exceeds 300. Based on these observations, the swarm size was set to 60 and the iteration number was set to 300 in all subsequent experiments to ensure stability and reproducibility of the optimization results. Through the local behaviors of individual fish and group collaboration, it gradually approximates the global optimal solution. Its core logic can be summarized as follows:

Individual behaviors: Each fish searches for locally optimal solutions through “foraging”, avoids isolation via “swarming”, and follows the optimal individual in the group through “following”. If all these behaviors fail, it performs “random swimming” to explore new regions.

Group collaboration: After each iteration, the “global optimal solution” is updated to guide the entire fish school to converge towards a more optimal region.

Optimization objective: Minimize the RMSE between the kernel density estimation value and the data histogram density” calculated by the objective function, and finally output the parameters c and α that minimize the RMSE.

Substitute the optimized parameters c and α into the bandwidth formula to obtain the final expression of AFSO-AKDE, as shown in Equation 9:

$$\hat{f}\left(x\right)=\frac{1}{\sqrt{2\pi }n\lambda c_{opt}\hat{\sigma }n^{‐\alpha_{opt}}}\sum_{i=1}^{n}e^{{\left[‐\frac{1}{2}\left(\frac{x‐x_{i}}{\lambda c_{opt}\hat{\sigma }n^{‐\alpha_{opt}}}\right)\right]}^{2}}$$ (9)

The quantitative results of the parameter sensitivity analysis are summarized in Table 1.

**TABLE 1:** Sensitivity analysis of AFSO parameters on RMSE.

Swarm Size	Iterations	RMSE
20	100	0.000412
40	200	0.000221
60	300	0.000156
80	300	0.000152
100	500	0.000150

The results indicate that the reduction in RMSE, becomes negligible when the swarm size exceeds 60 and the iteration number exceeds 300, confirming the robustness of the selected parameters.

#### Modeling process of β-decay energy spectrum probability density distribution based on AFSO-AKDE

**FIGURE 1:**
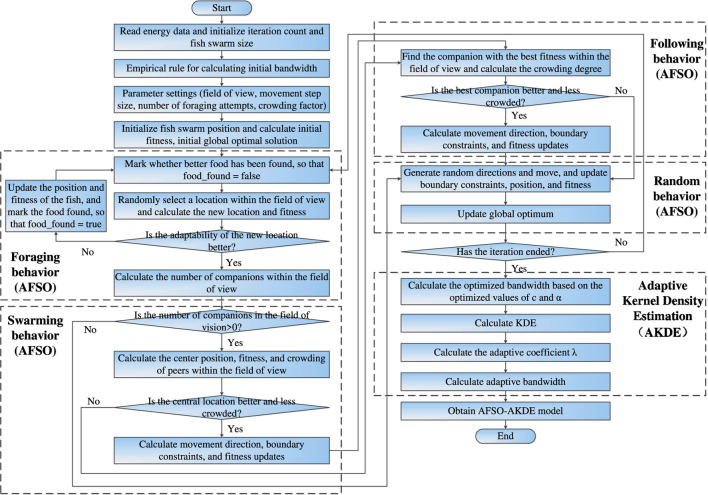
Flowchart of β energy spectrum probability density distribution modeling based on AFSO-AKDE.

The modeling process of β-decay energy spectrum probability density distribution based on AFSO-AKDE proposed in this paper is shown in Figure 1, which can be divided into three core stages: data preprocessing, AFSO optimization stage, and AKDE modeling stage. The specific process is as follows:

#### Data preprocessing

The emitted β spectral data from radioisotope source configurations are read and initialized, forming standardized spectral inputs for subsequent source-side probability density reconstruction.

#### AFSO optimization stage

The initialized data is input into the Artificial Fish Swarm Optimization (AFSO) module, and the effectiveness of foraging, swarming, and following behaviors is determined in sequence—if a behavior is successful, the position and fitness of the fish school are updated; if all three behaviors fail, a random behavior is executed. After completing the behavior execution, it is judged whether the iteration is over; if not, the above behavior determination and update process is repeated until the iteration meets the termination condition.

#### AKDE modeling stage

After the iteration ends, the optimized bandwidth is calculated based on the parameters ( c and α ) obtained by AFSO optimization. KDE calculation, solution of the adaptive coefficient λ , and determination of the adaptive bandwidth are completed in sequence. Finally, the AFSO-AKDE model is constructed, and the modeling of the β energy spectrum probability density distribution is completed. The measured computational time per iteration under different sample sizes is summarized in Table 2.

**TABLE 2:** Computational time per iteration under different sample sizes.

Sample size	Time per iteration (s)
1 × 10^4^	0.12
5 × 10^4^	0.67
1 × 10^5^	1.36

The results in Table 2 show that the computation time per iteration increases with the sample size in a consistent manner. When the sample size increases from 1 × 10^4^ to 5 × 10^4^, the time per iteration increases from 0.12 s to 0.67 s, indicating a clear growth trend associated with the increased number of kernel evaluations. When the sample size further increases to 1 × 10^5^, the time per iteration reaches 1.36 s, reflecting the cumulative computational cost introduced by pairwise density estimation. The observed scaling behavior is consistent with the theoretical complexity of the algorithm. Under the selected parameter settings, the total runtime remains bounded due to the limited number of iterations required for convergence, and the optimization process terminates within a stable computational time range. These results indicate that the proposed method maintains predictable computational behavior with respect to sample size, supporting its applicability in offline spectral modeling scenarios.

### Description of β energy spectrum data

**FIGURE 2:**
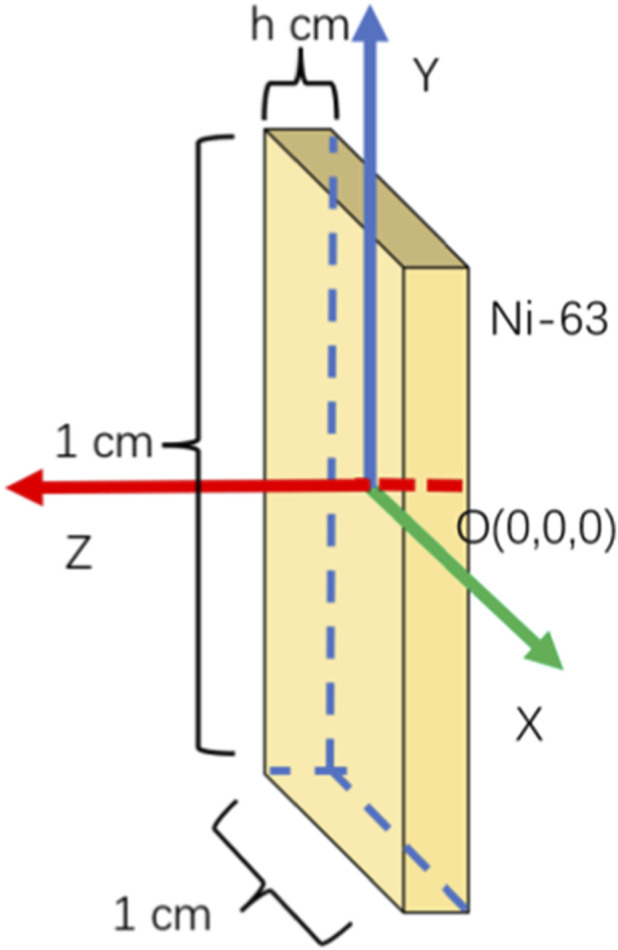
Radiation source decay model diagram in Geant4.

The Geant4 simulation is configured to generate emitted β spectra from candidate radioisotope source layers under controlled physical conditions, where a 1 cm × 1 cm×2 μm 63Ni source is constructed as a representative source structure for energy spectrum generation, as shown in Figure 2. The number of simulated particles was 10^6^, and the physical processes selected included “G4EmStandardPhysics()”, “G4DecayPhysics()”, and “G4RadioactiveDecayPhysics()”, covering the main interaction processes of β particles in semiconductors. In addition, detailed physics configuration parameters were specified to ensure the reproducibility of the simulation. The production cut for secondary particles was set to 1 μm for electrons and photons, ensuring accurate tracking of low-energy interactions. The energy range of particle transport was defined from 100 eV to 1 MeV to fully cover the β-decay spectrum of ^63^Ni. The step limitation was controlled through the default multiple scattering model to maintain numerical stability during transport simulation. The geometry configuration consisted of a uniform source layer with planar structure and vacuum boundary conditions, avoiding boundary-induced artifacts. All simulations were conducted under fixed random seed initialization to ensure consistent statistical sampling across repeated runs. The simulated interaction processes define the energy release and transport behavior within source materials, forming physically consistent spectral outputs associated with radioisotope energy conversion environments. The resulting energy data represent emitted β spectra from the radioisotope source layer and are treated as input spectral samples for source-side characterization in betavoltaic energy conversion modeling. When the radioactive source is ^63^Ni, its decay equation is shown in Equation 10:

N2863i→C2963u+e‐10+v¯e (10)

### Fitting evaluation metrics

To evaluate the goodness-of-fit of the probability density distribution function, three evaluation metrics—MAE (Hui et al., 2025), RMSE (Aly et al., 2024), and R^2^ (Ahmed et al., 2025)—are adopted to verify the effectiveness of the model. Smaller values of RMSE and MAE indicate higher accuracy of the model, while a R^2^ value closer to 1 reflects a better fitting degree of the model. The calculation formulas for the three evaluation metrics are given in Equations 11–13:

$$MAE=\frac{100}{n}\sum_{i=1}^{n}\left|\frac{\hat{f}\left(x_{i}\right)‐y\left(x_{i}\right)}{y\left(x_{i}\right)}\right|$$ (11)

$$RMSE=100\times \sqrt{\frac{1}{n}\sum_{i=1}^{n}{\left(\frac{\hat{f}\left(x_{i}\right)‐y\left(x_{i}\right)}{y\left(x_{i}\right)}\right)}^{2}}$$ (12)

$$R^{2}=\sqrt{\frac{\sum_{i=1}^{n}{\left[\hat{f}\left(x_{i}\right)‐\bar{y}\left(x_{i}\right)\right]}^{2}}{\sum_{i=1}^{n}{\left[y\left(x_{i}\right)‐\bar{y}\left(x_{i}\right)\right]}^{2}}}$$ (13)

In the formula: i = 1, 2, ., n, where n is the number of sample sequences; $y\left(x_{i}\right)$ is the ordinate of the i-th histogram; $\hat{f}\left(x_{i}\right)$ is the function value corresponding to the fitted probability density function; $\bar{y}\left(x_{i}\right)$ is the average value of the ordinate values of the histogram. MAE measures the average error magnitude and is insensitive to outliers; RMSE amplifies the impact of larger errors and is sensitive to outliers. Using both together can comprehensively evaluate the model’s performance under both overall fit and large bias conditions.

## Results and discussion

### Spectral distribution characteristics of ^63^Ni radioactive sources with different thicknesses

To analyze the impact of source engineering parameters on spectral distribution, ^63^Ni is selected as the research object, where source thickness is varied across 2μm, 0.2μm, and 0.02 μm to construct corresponding probability density histograms. Source thickness is treated as a key design parameter because it governs self-absorption behavior and energy-spectrum shaping within radioisotope energy conversion systems. The decay energy spectrum of ^63^Ni at different thicknesses were modeled using the AFSO-AKDE method, the normal distribution, and the traditional KDE method.

**FIGURE 3:**
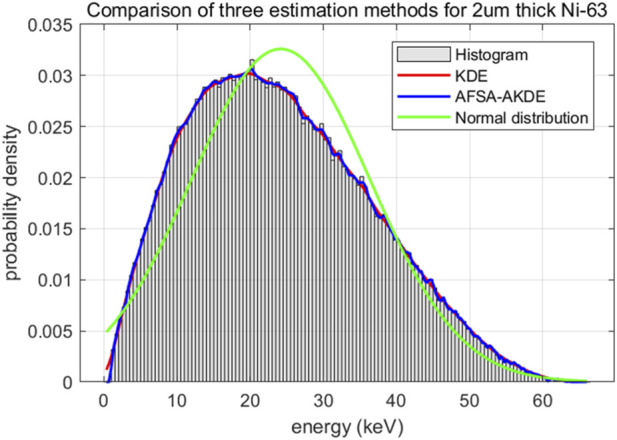
Comparison of fitted probability density curves for 2 μm-thick ^63^Ni.

As shown in Figure 3, the fitting effects of three methods on the energy spectrum of 2 μm-thick ^63^Ni material are compared. The curve obtained by the AFSA-AKDE method can better capture the detailed characteristics of the data distribution, especially exhibiting an obvious asymmetric multimodal structure in the 10–30 keV energy range, which is more consistent with the complex energy distribution of actual physical processes. Although the traditional KDE method also yields a relatively smooth curve, it is less clear than AFSA-AKDE in terms of peak shape details. The histogram is significantly affected by binning, showing an obvious step-like pattern and weak ability to express details. Parameter estimation based on the normal distribution is clearly inapplicable here, as it cannot describe the multimodal and asymmetric characteristics of the actual energy distribution, indicating that the energy release of ^63^Ni at this thickness has obvious non-normal characteristics.

**FIGURE 4:**
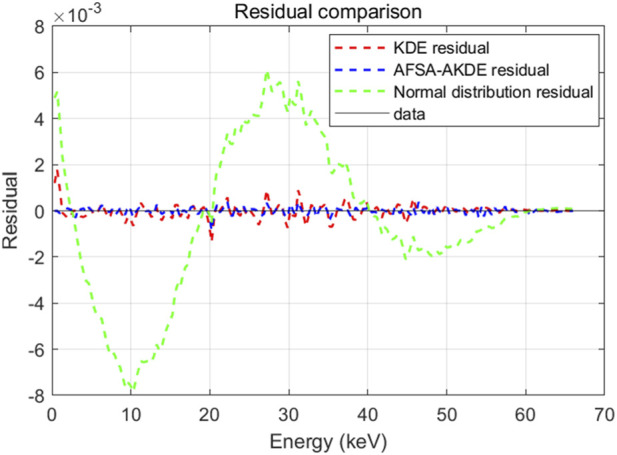
Residual comparison of different probability density models for 2 μm-thick ^63^Ni.

Figure 4 presents a comparison of residuals from three probability density models for the energy spectrum of 2 μm-thick ^63^Ni. The residuals of both KDE and AFSA-AKDE fluctuate around the zero line, with amplitudes significantly smaller than those of the normal distribution residuals. The AFSA-AKDE residuals show the smallest fluctuation amplitude and the highest concentration, while KDE residuals are slightly larger, indicating that KDE is slightly inferior in capturing distribution details. The AFSA-AKDE method yields the smallest residuals and the highest stability when fitting the ^63^Ni energy spectrum, further verifying its superiority in handling complex spectral data.

According to the comparison of quantitative evaluation metrics for the three probability density distribution models of 2 μm-thick ^63^Ni in Table 3, the AFSA-AKDE method exhibits the best performance: its MAE (0.0116%) and RMSE (0.0156%) are the smallest, and its R^2^ (0.9998792) is closest to 1, indicating that the model has extremely low fitting error and the highest degree of agreement with the data, far outperforming KDE and the normal distribution. It can be concluded that if the prior distribution is incorrectly selected, the parameter estimation method can hardly achieve satisfactory modeling accuracy.

**TABLE 3:** Comparison of evaluation metrics for probability density distribution models of 2 μm-thick^63^Ni.

Evaluation metric	KDE	AFSA-AKDE	Normal distribution
MAE (%)	0.0204	0.0116%	0.2561
RMSE (%)	0.0314	0.0156	0.3365
R^2^	0.999151	0.9998792	0.902664

To further validate the performance of the proposed method, additional comparisons were conducted with recent adaptive KDE variants reported in the literature, including diffusion-based KDE and regularized variable KDE. The comparative results are summarized in Table 4.

**TABLE 4:** Comparison with state-of-the-art adaptive KDE methods for ^63^Ni (2 μm).

Method	MAE	RMSE	R^2^
Diffusion KDE (%)	0.0189	0.0276	0.999201
Regularized KDE (%)	0.0172	0.0241	0.999356
AFSA-AKDE (%)	0.0116	0.0156	0.9998792

The results show that AFSA-AKDE, maintains lower error and higher fitting consistency compared with recent adaptive KDE, approaches.

**FIGURE 5:**
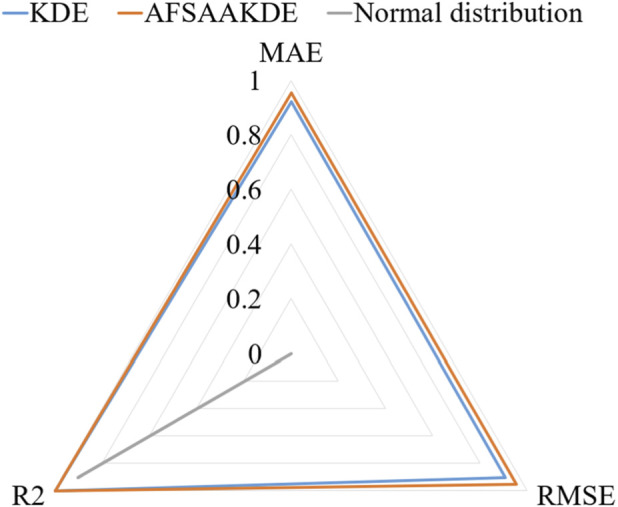
Radar chart of standardized metrics for 2 μm-thick ^63^Ni.

Figure 5 presents a comparison of standardized metrics in the radar chart shows that AFSA-AKDE performs optimally across all three indicators: its standardized values of MAE, RMSE, and R^2^ are all closest to 1 (i.e., the three vertices in the chart), forming the largest triangular area. This indicates that AFSA-AKDE has the best comprehensive performance, the smallest error, and the highest fitting degree.

**FIGURE 6:**
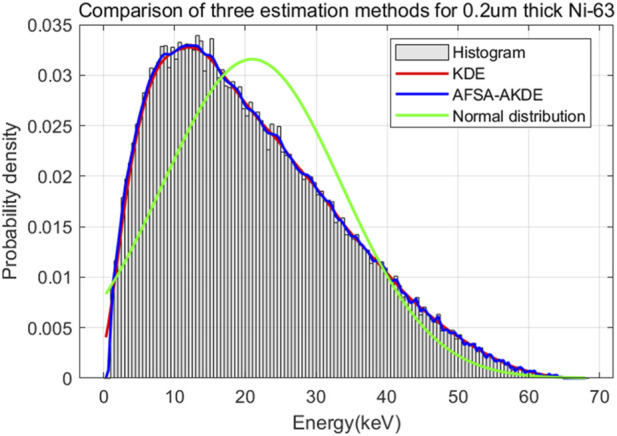
Comparison of fitted probability density curves for 0.2 μm-thick ^63^Ni.

**FIGURE 7:**
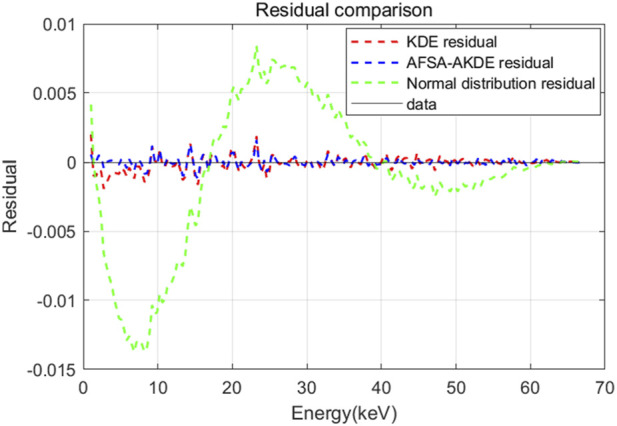
Residual comparison of different probability density models for 0.2 μm-thick ^63^Ni.

Figure 6 shows the energy histogram of 0.2 μm-thick ^63^Ni material and compares the fitting effects of three probability density estimation methods on the energy spectrum. The AFSA-AKDE method achieves the best fitting effect, with the trend most consistent with the original histogram. The residual evaluation plot is presented in Figure 7 For thinner ^63^Ni samples, the AFSA-AKDE method remains significantly superior to KDE and the normal distribution in controlling fitting errors and maintaining stability.

**FIGURE 8:**
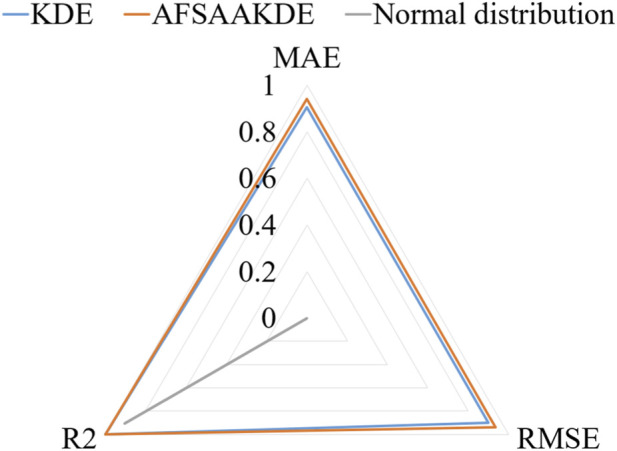
Radar chart of standardized metrics for 0.2 μm-thick ^63^Ni.

The results of fitting accuracy evaluation are presented in Table 5 AFSA-AKDE performs optimally across all metrics: its MAE (0.0223%) and RMSE (0.0340%) are the smallest, and its R^2^ (0.999114) is closest to 1, indicating the lowest fitting error and the strongest ability to explain data variability. For intuitive evaluation, a radar chart is plotted as shown in Figure 8 AFSA-AKDE’s values for the three standardized metrics (MAE, RMSE, and R^2^) are all closest to 1, forming the largest convex triangle, which demonstrates the smallest error, the highest goodness-of-fit, and a significant lead in comprehensive performance.

**TABLE 5:** Comparison of evaluation metrics for probability density distribution models of 0.2 μm-thick^63^Ni.

Evaluation metric	KDE	AFSA-AKDE	Normal distribution
MAE (%)	0.0364	0.0223	0.3840
RMSE (%)	0.0539	0.0340	0.5342
R^2^	0.997774	0.999114	0.781526

**FIGURE 9:**
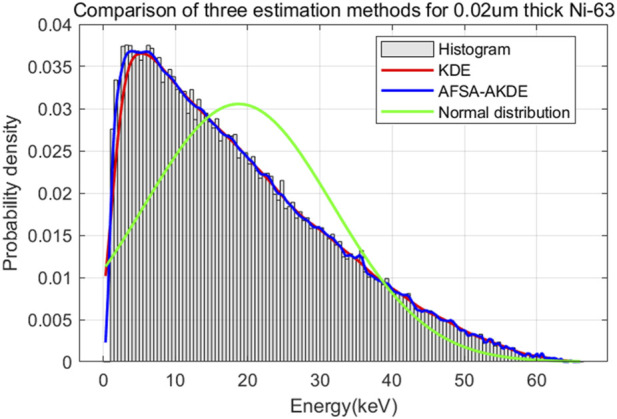
Comparison of fitted probability density curves for 0.02 μm-thick ^63^Ni.

**FIGURE 10:**
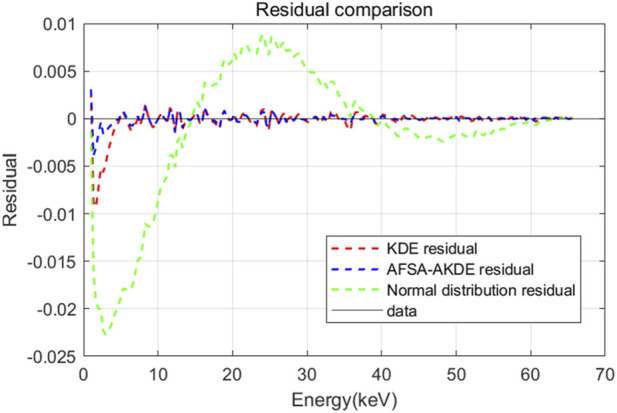
Residual comparison of different probability density models for 0.02 μm-thick ^63^Ni.

**FIGURE 11:**
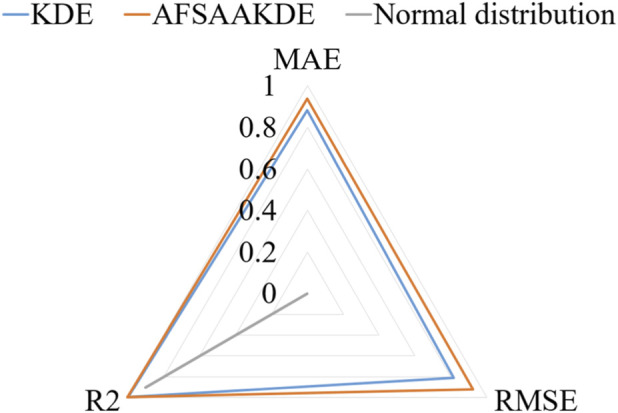
Radar chart of standardized metrics for 0.02 μm-thick ^63^Ni.

Figure 9 presents the energy histogram of 0.02 μm-thick ^63^Ni material and compares the fitting effects of three probability density estimation methods on the energy spectrum. The AFSA-AKDE method achieves the best fitting effect, with the trend most consistent with the original histogram. The residual evaluation plot is shown in Figure 10 For thinner ^63^Ni samples, the AFSA-AKDE method remains significantly superior to KDE and the normal distribution in controlling fitting errors and maintaining stability. For quantitative evaluation, MAE, RMSE, and R^2^ were calculated as shown in Table 6 AFSA-AKDE yields the lowest MAE (0.0308%) and RMSE (0.0579%), with its R^2^ (0.997771) closest to 1, indicating that it maintains the lowest fitting error and the highest explanatory power even for ultra-thin samples. The radar chart in Figure 11 also intuitively shows that AFSA-AKDE performs best across all three dimensions: its standardized values of MAE, RMSE, and R^2^ are closest to 1, forming the largest triangular area that is closest to the outer edge, demonstrating optimal comprehensive fitting performance.

**TABLE 6:** Comparison of evaluation metrics for probability density distribution models of 0.02 μm-thick^63^Ni.

Evaluation metric	KDE	AFSA-AKDE	Normal distribution
MAE (%)	5.76	3.08	48.12
RMSE (%)	13.64	5.79	73.76
R^2^	0.987656	0.997771	0.638829

### Spectral distribution characteristics of different β radioactive source materials

To analyze the spectral distribution characteristics across candidate source materials, the thickness is fixed at 0.2 μm, and probability density histograms are generated for different β-emitting radioisotopes, including ^63^Ni, ^35^S, and ^14^C, as candidate source materials for betavoltaic applications. The emitted β spectra from different candidate source materials at identical thickness are modeled to evaluate spectral representation consistency under varying radioisotope selections within energy conversion scenarios.

**FIGURE 12:**
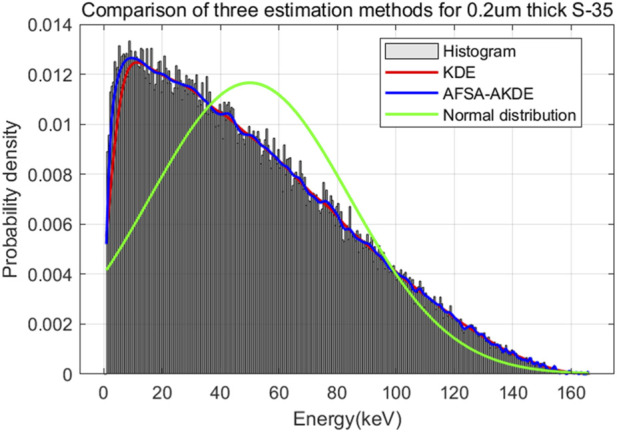
Comparison of fitted probability density curves for 0.2 μm-thick ^35^S.

**FIGURE 13:**
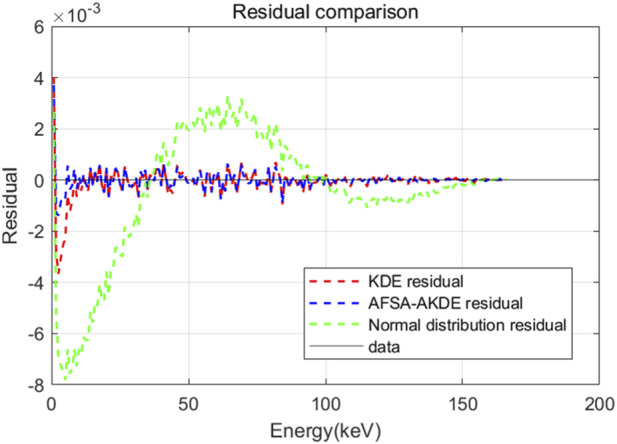
Residual comparison of different probability density models for 0.2 μm-thick ^35^S.

**FIGURE 14:**
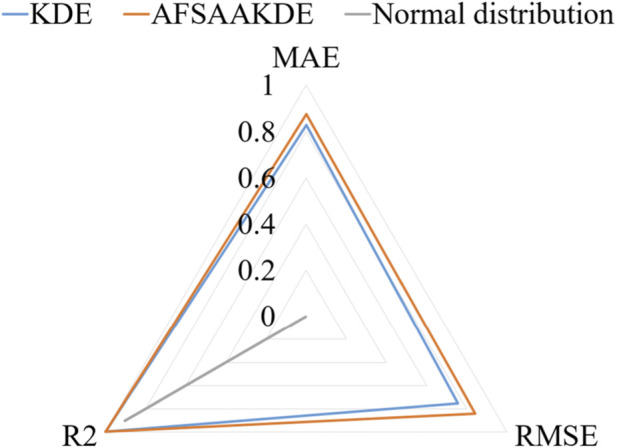
Radar chart of standardized metrics for 0.2 μm-thick ^35^S.

Figure 12 presents the energy histogram of 0.2 μm-thick ^35^S material and compares the fitting effects of three probability density estimation methods on the energy spectrum. The AFSA-AKDE method achieves the best fitting effect, with the trend most consistent with the original histogram. The residual evaluation plot is shown in Figure 13. For the 0.2 μm-thick ^35^S sample, the AFSA-AKDE method remains significantly superior to KDE and the normal distribution in controlling fitting errors and maintaining stability. For quantitative evaluation, MAE, RMSE, and R^2^ were calculated as shown in Table 7 AFSA-AKDE yields the lowest MAE (0.0212) and RMSE (0.0397), with its R^2^ (0.99149) closest to 1, indicating that it maintains the lowest fitting error and the highest explanatory power for ^35^S samples. The radar chart in Figure 14 also intuitively shows that AFSA-AKDE performs best across all three dimensions: its standardized values of MAE, RMSE, and R^2^ are closest to 1, forming the largest triangular area that is closest to the outer edge, demonstrating optimal comprehensive fitting performance.

**TABLE 7:** Comparison of evaluation metrics for probability density distribution models of 0.2 μm-thick ^35^S.

Evaluation metric	KDE	AFSA-AKDE	Normal distribution
MAE (%)	0.0294	0.0212	0.1702
RMSE (%)	0.0617	0.0397	0.2531
R^2^ (%)	0.979480	0.99149	0.654405

**FIGURE 15:**
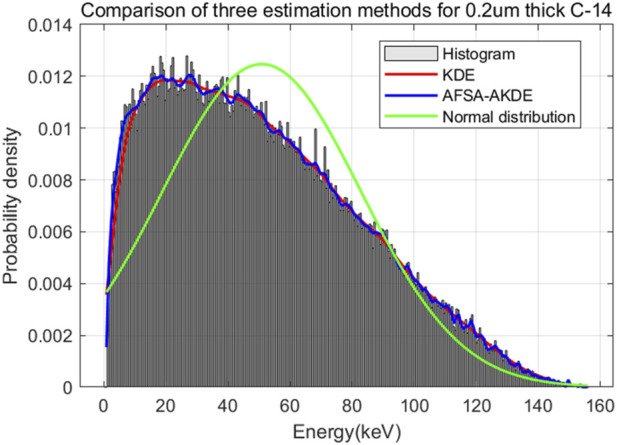
Comparison of fitted probability density curves for 0.2 μm-thick ^14^C.

**FIGURE 16:**
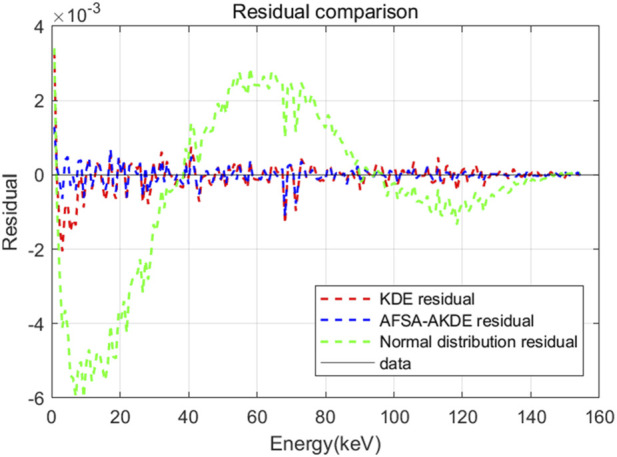
Residual comparison of different probability density models for 0.2 μm-thick ^14^C.

**FIGURE 17:**
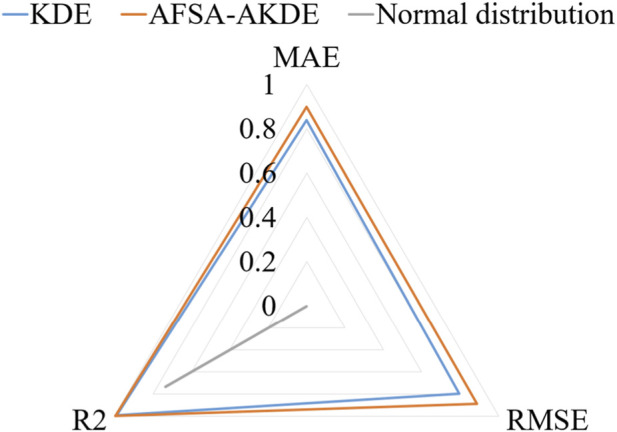
Radar chart of standardized metrics for 0.2 μm-thick ^14^C.

Figure 15 presents the energy histogram of 0.2 μm-thick ^14^C material and compares the fitting effects of three probability density estimation methods on the energy spectrum. The AFSA-AKDE method achieves the best fitting effect, with the trend most consistent with the original histogram. The residual evaluation plot is shown in Figure 16. For the 0.2 μm-thick ^14^C sample, the AFSA-AKDE method remains significantly superior to KDE and the normal distribution in controlling fitting errors and maintaining stability. For quantitative evaluation, MAE, RMSE, and R^2^ were calculated as shown in Table 8. AFSA-AKDE yields the lowest MAE (0.0158%) and RMSE (0.0243%), with its R^2^ (0.996687) closest to 1, indicating that it maintains the lowest fitting error and the highest explanatory power for ^14^C samples. The radar chart in Figure 17 also intuitively shows that AFSA-AKDE performs best across all three dimensions: its standardized values of MAE, RMSE, and R^2^ are closest to 1, forming the largest triangular area that is closest to the outer edge, demonstrating optimal comprehensive fitting performance.

**TABLE 8:** Comparison of evaluation metrics for probability density distribution models of 0.2 μm-thick ^14^C.

Evaluation metric	KDE	AFSA-AKDE	Normal distribution
MAE (%)	0.0250	0.0158	0.1547
RMSE (%)	0.0443	0.0243	0.2172
R^2^	0.988959	0.996687	0.734860

### Implications for betavoltaic source design and energy conversion modeling

To characterize the distribution characteristics of the β energy spectrum under the combined effects of source structure and nuclide selection, and to establish the correlation between statistical modeling results and energy conversion behavior, emission spectra under different source conditions were uniformly characterized. Source conditions consist of two types of variables: one is the source layer structure formed by ^63^Ni at thicknesses of 2 μm, 0.2 μm, and 0.02 μm, reflecting the modulation effect of source thickness variations on particle transport paths and energy deposition distribution; the other is the ^63^Ni, ^35^S, and ^14^C nuclide systems under the same 0.2 μm thickness condition, characterizing the energy spectrum differences caused by different β-decay mechanisms. Based on this, three statistical quantities—spectral mean, skewness, and high-energy effective proportion—were extracted from the reconstructed probability density function. The spectral mean describes the overall energy level, skewness reflects the asymmetric structure of the energy spectrum, and the high-energy effective proportion characterizes the proportion of energy contribution exceeding a set energy threshold. All data are derived from emission energy spectrum results obtained under a unified physics process setting in the Geant4 simulation environment. The relevant statistical results are shown in Table 9.

**TABLE 9:** Characteristic parameters of β-spectrum and the effective proportion of high energy under different source conditions.

Source condition	Mean emitted energy (keV)	Skewness	Effective high-energy fraction
^63^Ni 2 μm	17.842	0.913	0.182
^63^Ni 0.2 μm	21.376	0.746	0.268
^63^Ni 0.02 μm	24.915	0.588	0.341
^35^S 0.2 μm	48.237	0.521	0.563
^14^C 0.2 μm	36.104	0.637	0.417

Table 9 shows that as the thickness of the ^63^Ni source layer increases from 0.02 μm to 2 μm, the spectral mean decreases from 24.915 keV to 17.842 keV, the effective proportion of high-energy particles decreases from 0.341 to 0.182, and the skewness increases from 0.588 to 0.913. This indicates that as particles travel a longer path within the source layer, high-energy components continuously decay and shift to lower energy regions, thus enhancing the asymmetry of the energy spectrum. This change reflects the reshaping effect of self-absorption on the energy distribution. Under the same thickness conditions, significant differences are observed among different nuclides. The spectral mean of 35S reaches 48.237 keV with an effective proportion of high-energy particles of 0.563, while 14C corresponds to 36.104 keV and 0.417, and ^63^Ni to 21.376 keV and 0.268. This indicates that the nuclide decay energy level structure directly determines the contribution ratio of high-energy particles and the overall energy level. The difference in skewness further reflects the structural differences in the spectral distribution under different energy release processes. The above results indicate that the source layer thickness controls the energy decay path, and the nuclide type determines the initial energy distribution. The two work together to form an energy spectrum structure with a clear physical orientation.

## Discussion

This study addresses the absence of a unified high-precision framework for β-decay spectral characterization under asymmetric distribution conditions by explicitly linking statistical representation to the underlying physical mechanisms of radioisotope energy materials. In β-emitting source materials, variations in source thickness directly alter particle transport paths, while self-absorption causes partial energy dissipation prior to emission, thereby shifting spectral weight toward lower-energy regions and compressing the high-energy tail. At the same time, differences in radionuclide endpoint energies define the upper energy bound and overall structural extent of the spectrum, leading to systematic changes in skewness, tail behavior, and effective energy-release characteristics. From this perspective, the probability density function is not merely a mathematical description of spectral data, but rather a theoretical representation of decay structure, transport dynamics, and energy-release behavior. Accordingly, nonparametric density estimation may be regarded as a data-driven carrier of physically constrained spectral information for radioisotope energy systems.

The observed fitting stability and error convergence arise from the coordinated interplay between adaptive bandwidth modulation and global optimization. The adaptive bandwidth mechanism preserves local structural resolution in regions with sharp density variation, whereas the artificial fish swarm optimization algorithm constrains the global parameter space to maintain consistency, smoothness, and robustness across the entire spectrum. This balance between local sensitivity and global regularity is particularly important for β-decay spectra, whose asymmetric and multiscale features often challenge conventional parametric representations. From the perspective of energy-conversion applications, these spectral variations also impose practical engineering constraints. Increasing source thickness generally reduces the fraction of high-energy emitted particles, whereas radionuclide selection determines the available energy range, penetration depth, and deposition characteristics. Because source-spectrum accuracy propagates directly into transport calculations, energy-deposition analysis, and device-level performance prediction, even small representation errors may accumulate across coupled physical processes. Therefore, the ultimate performance of the present framework depends not only on sample resolution and kernel expressiveness, but also on the degree of physical abstraction embedded in the modeling process. This suggests that future incorporation of transport-informed constraints, conservation laws, or physics-guided regularization could further enhance representational completeness and physical interpretability.

Within the broader landscape of theoretical research on energy materials, the proposed method operates at the source-term modeling level and complements first-principles calculations, multiscale transport simulations, and device-scale analyses. Unlike atomistic approaches that focus on electronic structure or microstructural mechanisms, the present framework reconstructs β-decay source spectra at the statistical level and thereby provides an efficient interface between radiation source characterization and higher-level energy-conversion modeling. Its significance lies not only in improving fitting accuracy, but also in establishing a scalable data-driven paradigm that links physical mechanisms, statistical representation, and engineering-oriented modeling through structured optimization. In this sense, the method offers a practical theoretical tool for source-side characterization, model coupling, and performance-oriented analysis of radioisotope energy materials, and may support future studies on source optimization, multiphysics integration, and cross-scale modeling in betavoltaic energy-conversion systems.

## Conclusion

This study addresses the challenge of accurately characterizing complex β-decay energy spectra by developing an adaptive kernel density estimation (AKDE) framework driven by Artificial Fish Swarm Optimization (AFSA). By integrating data preprocessing, swarm-intelligence-based parameter tuning, and adaptive bandwidth modeling, the proposed method achieves high-precision probability density reconstruction while maintaining global spectral consistency. Experimental validation demonstrates the model’s high fidelity across various radionuclides: for a 2 μm ^63^ *Ni* sample, the model attained an MAE of 0.0116%, an RMSE of 0.0156%, and an $R^{2}$ of 0.9998792. Similar robustness was observed for a 0.2 μm ^14^C sample (R^2^ = 0.996687) and a 0.2 μm ^35^S sample (R^2^ = 0.99149). These consistently low error metrics across varying thicknesses and isotopes confirm the method’s efficacy in capturing multi-peak, asymmetric, and long-tailed spectral features.

Beyond statistical fitting, the proposed framework establishes a theoretically grounded source-term characterization strategy for radioisotope energy materials. It explicitly bridges mathematical spectral representation with the underlying physical mechanisms of radioactive decay, particle transport, and energy deposition. This capability provides high-precision inputs for source-layer design, radiation transport analysis, and performance-oriented modeling in betavoltaic systems. Moreover, the method enables efficient information transfer across modeling scales, serving as a data-driven interface between source-side spectral reconstruction and system-level multiphysics simulations. Future work will focus on incorporating transport-informed constraints and conservation-aware architectures to further enhance the physical consistency of the model. In addition, advanced machine learning strategies will be explored to improve generalization capability and modeling reliability in increasingly complex energy-conversion scenarios.

## Data Availability

The original contributions presented in the study are included in the article/supplementary material, further inquiries can be directed to the corresponding author.
